# How Can Satellite DNA Divergence Cause Reproductive Isolation? Let Us Count the Chromosomal Ways

**DOI:** 10.1155/2012/430136

**Published:** 2012-01-29

**Authors:** Patrick M. Ferree, Satyaki Prasad

**Affiliations:** ^1^W. M. Keck Science Department, The Claremont Colleges, Claremont, CA 91711, USA; ^2^Department of Molecular Biology and Genetics, Cornell University, Ithaca, NY 14853, USA

## Abstract

Satellites are one of the most enigmatic parts of the eukaryotic genome. These highly repetitive, noncoding sequences make up as much as half or more of the genomic content and are known to play essential roles in chromosome segregation during meiosis and mitosis, yet they evolve rapidly between closely related species. Research over the last several decades has revealed that satellite divergence can serve as a formidable reproductive barrier between sibling species. Here we highlight several key studies on Drosophila and other model organisms demonstrating deleterious effects of satellites and their rapid evolution on the structure and function of chromosomes in interspecies hybrids. These studies demonstrate that satellites can impact chromosomes at a number of different developmental stages and through distinct cellular mechanisms, including heterochromatin formation. These findings have important implications for how loci that cause postzygotic reproductive isolation are viewed.

## 1. Introduction

Decades ago when researchers began purifying DNA from eukaryotes using cesium chloride gradients, they observed bands of DNA that were distinct from the major genomic bands. The sequences comprising these ancillary bands were named satellites—a term from Greek meaning “followers of a superior entity”—and were found to separate from the other sequences due to their adenosine- and thymine-rich base pair compositions. Since their discovery, satellites have proven to be one of the most intriguing parts of the genome, owing to their high abundance, rapid evolutionary change, and a growing body of evidence indicating that they can impact speciation.

The abundance of satellites varies widely in eukaryotic genomes, from effectively 0% in yeast species such as *Schizosaccharomyces pombe* to 25–50% or more in Drosophila and mammalian species [2–4]. Individual satellite monomers also vary dramatically in their monomer length, from the *D. melanogaster* pentameric monomer, AATAT, to more complex monomers such as the 972-bp centromeric satellite in the Indian muntjac [5]. Satellite monomers such as these are organized into arrays, or blocks, of tens to thousands of tandem copies located in the centromeres, the telomeres, and their surrounding regions. Indeed, the Y chromosome in many higher eukaryotes consists almost entirely of satellites. Despite their abundance, satellites are nonprotein coding and were therefore hypothesized to be genomic “junk” [6] or even selfish genetic elements [7]. Contrary to the former idea, the chromosomal regions consisting of satellites are now known to play important but incompletely understood roles in the structure, stability, and segregation of the chromosomes [8–10]. The idea that satellites are selfish elements remains to be determined.

**Figure 1:**
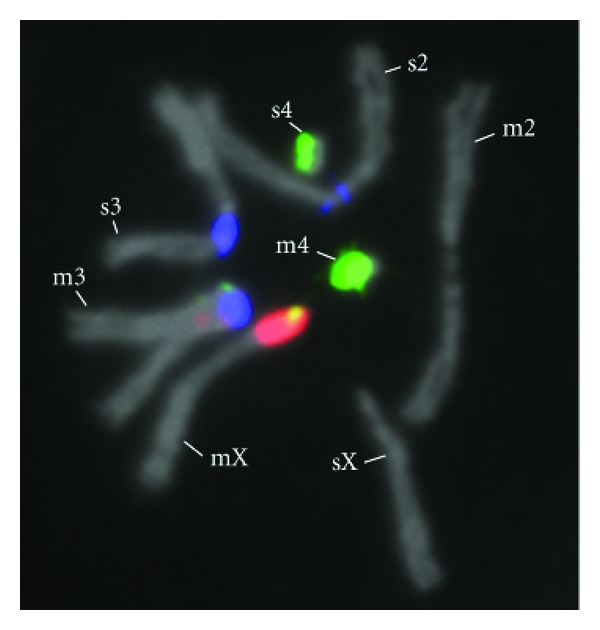
Satellite block divergence between *Drosophila melanogaster* and *D. simulans*. Each chromosome pair, consisting of one homologous chromosome from each species, shows remarkable satellite differences: the *D. melanogaster* X contains a large block of the 359-bp satellite (red) and some AATAT (green) while the *D. simulans* X contains neither of these specific satellite monomers; dodeca satellite (blue) is present on the *D. melanogaster* 2nd chromosome and absent on the *D. simulans* 2nd chromosome; large regions of dodeca satellite are present on the 3rd chromosomes of both species, but only *D. melanogaster* 3rd chromosome has small regions of AATAT (green) and a small region of 359-bp variant (also red); AATAT satellite (green) is more abundant and distributed widely across the *D. melanogaster* 4th chromosome while the *D. simulans* 4th chromosome contains two primary regions of AATAT, which cannot be fully seen in this image, and in smaller amounts. Chromosomes were prepared from mitotic brain cells of hybrid larvae and stained by fluorescence in situ hybridization (FISH) as previously described [1].

 Given the high abundance of satellites and their involvement in chromosome behavior, it is intriguing that these sequences make up one of the most rapidly evolving parts of the genome. Studies conducted over the last four decades have revealed large disparities in satellite abundance between closely related species within insect, mammal, and plant groups [11–16]. Owing to rapid expansions and contractions in copy number, specific satellite blocks may be either severely reduced in size or altogether absent in close relatives (Figure 1) [1, 13, 17, 18]. Additionally, the monomers of some complex satellites can differ in sequence composition between closely related species at levels higher than the average genome-wide divergence [19]. However, certain regions of some centromere satellite monomers and even whole monomers are highly conserved, perhaps out of necessity to maintain their interactions with centromere-associated proteins [20–22].

 Various mechanisms, including unequal recombination, gene conversion events, and replication slippage, have been proposed to explain how individual satellite blocks can evolve rapidly [23, 24]. These processes can generate satellite blocks of widely varying sizes (i.e., those containing different copy numbers) within a given species. This variation can influence chromosome dynamics and individual fitness in a number of different ways. For example, large blocks of the *D. melanogaster* Responder (*Rsp*) satellite can be deleterious under certain genetic conditions. Located on the *D. melanogaster* 2nd chromosome, the *Rsp* block is highly variable, ranging from ~10 to over 3,000 monomers per block among individuals [25]. Second chromosomes carrying large *Rsp* blocks are targeted for destruction during spermatogenesis if the other 2nd chromosome carries a selfish allele of the Segregation Distorter (*Sd*) gene and a small *Rsp* block. This effect results in the loss of half the sperm—those carrying the large *Rsp* block—and, thus, high transmission frequencies of the *Sd*-carrying chromosome. In contrast, variants of other satellite blocks may be functionally important for chromosome function and the fitness of the individual. One such case is the 359-bp satellite block on the X chromosome of *D. melanogaster*, which is located immediately adjacent to the rDNA locus and may play a role in regulating expression of the rDNA genes [26]. Finally, satellites can expand without affecting chromosome function. This trend appears to be true for satellites present on supernumerary B chromosomes, such as the Paternal Sex Ratio (PSR) chromosome in the jewel wasp, *Nasonia vitripennis* [27, 28]. Since this chromosome is not essential for the viability of its host, the satellites on them may be free from functional constraints and, therefore, able to expand and contract rapidly without effect.

These observations raise a compelling question—how can rapid changes in satellites affect the biology of their resident chromosomes and, ultimately, the organisms in which they reside? One context in which this question can be addressed is the impact of satellite divergence on interspecies hybrids. Early studies demonstrated that certain reproductively isolated species—that is, those that fail to produce fertile or viable hybrid offspring when they intermate—can exhibit large differences in composition and organization of their satellite blocks [1, 11–14]. These observations led to the suggestion that satellite divergence may contribute to speciation by causing reproductive isolation between species [11, 29]. Is there any validity to this idea, and if so, how might such an effect occur?

 In addressing these questions, we describe three general ways in which satellite differences between species could affect chromosome behavior in hybrids: (i) by disruption of chromosome pairing, (ii) by alteration of the chromatin structure of the satellites themselves or their surrounding sequences, or (iii) by involvement of satellites in meiotic or postmeiotic chromosome drive systems. We cite data from previous studies, primarily in Drosophila but also other organisms, that either support or argue against these possibilities. We also describe plausible molecular mechanisms that may underlie these effects. These examples provide new ways of viewing the types of loci that cause reproductive isolation and how they can evolve and operate at the molecular level in hybrids.

## 2. Disruption of Chromosome Pairing

One process that satellite divergence may affect in hybrids is homolog pairing, whereby similar sequences associate together in close proximity across homologous chromosomes. Pairing is a key aspect of meiosis, and much of what is known about pairing during meiosis derives from studies in *D. melanogaster*. During meiosis I in this organism, pairs of homologous chromatids align side by side at the metaphase plate before they segregate into daughter nuclei. The pairing of homologous sequences occurs before entry into meiosis and is ultimately important in Drosophila and other eukaryotes across the phyla for proper segregation of chromosomes and, therefore, the formation of functional gametes [30].

 There are, however, fundamental differences between male and female meiosis in flies that reflect to what degree satellite divergence may affect homolog pairing. In the pure species *D. melanogaster*, the involvement of repetitive sequences in pairing varies depending on the sex of the individual and the particular chromosome pair. For example, recombination occurs only in the female sex. Thus, synaptonemal complexes and chiasmata, or stable crossover junctions that help to hold the recombining homologs together before segregation, do not form in males [31]. The lack of these structures in males originally suggested that sequence specific interactions must instead dictate chromosome pairing in this sex [32, 33]. Years of work on this topic have shown that small “pairing sites” mediate homolog pairing in males. These sites include sequences found in the gene-containing regions of the autosomes and a single cluster of rDNA spacer repeats on the X and Y chromosomes [33, 34]. However, no data has been found to link satellite DNA or the pericentric regions where they are located with homolog pairing in male meiosis.

 In contrast to male flies, satellites may play an important role in meiotic homolog pairing in female flies. Experiments in which recombination, and thus, chiasmata are prevented from forming either through mutations abrogating recombination or through chromosomal inversions revealed that pairing occurs without these structures (reviewed in [35]). Additionally, the 4th chromosomes are largely achiasmatic. Thus, pairing in females is determined not by recombination-mediated structures but instead by sequence-specific interactions. Deletions of the satellite-containing X and 4th pericentric regions, but not the gene-containing regions, were shown to disrupt meiotic homolog pairing in females [35]. Thus, unlike in males, pericentric repetitive sequences may play a strong role in homolog pairing in females.

 The fact that the pericentric regions do not influence homolog pairing in pure species *D. melanogaster* males leads to the strong expectation that interspecies divergence of satellite DNA would not affect pairing in Drosophila hybrid males. However, the involvement of these regions in female meiosis legitimizes early speculation that substantial differences in satellites may inhibit meiotic homolog pairing in Drosophila hybrid females [29]. Is there any experimental evidence for these predictions? *D. melanogaster*/*D. simulans* hybrids of either sex normally do not produce gonads, thus precluding the analysis of homolog pairing in these individuals. In order to circumvent this problem, partial male hybrids—those carrying small chromosomal regions or single chromosomes from one species in the genetic background of the other species—were produced [36]. Of particular interest was one type of partial male hybrid containing both the *D. melanogaster* and *D. simulans* 4th chromosomes. These interspecific homologs were found to pair and segregate normally during meiosis [36] despite substantial differences in their satellite DNA content [13]. This result is consistent with the lack of involvement of repetitive sequences in meiotic homolog pairing in *D. melanogaster* pure species males.

 Currently, only a few other animal and plant hybrids have been examined. These analyses have focused primarily on the male sex, and while mispairing has been observed in some cases, the findings generally do not support a role of satellite divergence as a cause. In mice, male hybrids produced from *Mus musculus *and *M. poschiavinus* showed normal homolog pairing despite substantial, genome-wide differences in repetitive sequences [37]. In another case, *M. domesticus*/*M. spretus* male hybrids exhibited defective X-Y pairing [38]. The causal locus was mapped to a region near the cytological point of pairing between these chromosomes in the pure species. This finding suggested that a single pairing site, similar to the one that determines pairing of the X and Y in *D. melanogaster *males, is solely involved. In plants, crosses between species belonging to the Paeonia genus revealed incomplete homolog pairing in several different species combinations [39]. Because no major chromosomal inversions were found between these species, it was concluded that mispairing likely resulted from interspecies divergence of pairing genes. However, divergence of repetitive sequences was not discussed as formal possibility.

 Taken together, the above results suggest that satellite divergence does not affect meiotic homolog pairing in hybrids under certain species-, sex-, and chromosome-specific contexts. However, additional experiments are needed in other contexts, such as X or 4th homolog pairing in Drosophila hybrid females, in which there is a strong precedence for expecting such an effect. Studies employing specific mutations that allow *D. melanogaster*/*D. simulans* hybrid females to develop functional gonads [40, 41] will be helpful in more fully addressing the impact of satellite divergence on meiotic homolog pairing.

 Homolog pairing also occurs in the somatic tissues of Dipterans [42]. It has been proposed that somatic homolog pairing may play a role in the repair of double strand DNA breaks, the transitioning of premeiotic cells into meiosis, or transchromosome gene interactions [34, 42, 43]. Similar to meiotic pairing in females, pairing in somatic cells occurs between the pericentric regions in *D. melanogaster* [44]. What drives these interactions is not clear, but one possibility is high similarity of repetitive sequences between homologous chromosomes. This idea was argued against, however, by the results of one study in which a ~1.6 megabase pair block of AAGAG satellite located on the tip of the rearranged *D. melanogaster* 2nd chromosome, *bw* ^D^, was recombined onto the *D. simulans* 2nd chromosome and placed into the *D. simulans* genome [45]. In the *D. melanogaster* pure species, this satellite block associated with the pericentric region of the same 2nd chromosome, which also contains several blocks of AAGAG. When placed into the *D. simulans* genome, the *bw* ^D^-derived AAGAG block associated with the pericentric region on the 2nd chromosome of this species, despite the fact that it does not contain AAGAG satellite DNA. Moreover, the *bw* ^D^-derived AAGAG block did not associate with either of the *D. simulans* sex chromosomes, which do contain AAGAG satellite DNA. It was concluded from these results that pairing in somatic cells might not result from similarity of homologous sequences, but instead, through sequence-independent attractive forces between large regions of repetitive DNA.

 This conclusion may only partially explain somatic homolog pairing. Sequence-independent pairing alone would be expected to result in inappropriate associations of nonhomologous chromosomes during mitosis, and their missegregation, since all chromosomes in flies contain large amounts of repetitive sequences in their pericentric regions [11, 13]. A more likely scenario may be that both sequence-dependent and independent interactions govern pairing in somatic cells. Previous experiments have demonstrated that somatic pairing in the *D. melanogaster* pure species occurs at specific pericentric regions, such as the *Rsp* locus as well as AACAC and AAGAC satellite blocks [44]. Interestingly, the *Rsp* block is not present on the 2nd chromosome in *D. simulans *[46], and other pairing sequences may also be unique or substantially different between these species. Thus, the *D. simulans*/*D. melanogaster* hybrid is a promising system for taking advantage of these satellite differences in order to more fully explore the effects of satellite divergence on somatic homolog pairing.

## 3. Alteration of Chromatin Structure I: Satellite DNA/Protein Interactions

Another fundamental aspect of chromosome dynamics is the formation of chromosomes from chromatin. Occurring at entry into mitosis and meiosis, this process involves a number of structural proteins including Condensins and Topoisomerases [47]. These factors become distributed across the entire axes of the chromosomes as they condense at prophase. Other proteins, however, localize to discrete chromosomal regions, such as satellite blocks. For example, the *D. melanogaster* GAGA factor binds to AAGAG and AAGAGAG satellite monomers located in discrete regions on all of the chromosomes in this species [46]. GAGA factor and other satellite-binding proteins, such as Prod, are also transcription factors [48, 49].

 The nature of these satellite DNA/protein associations is not well understood. However, it has been proposed that satellite-binding transcription factors may play a role in bending or packaging satellite DNA [26, 50, 51]. This idea is supported by the observation that loss-of-function mutations in the gene encoding GAGA factor result in severe chromosome decondensation and segregation failure [52]. Additionally, this result is consistent with the fact that GAGA associates with the FACT complex, which together may play a more global role in chromatin packaging of repetitive sequences [53].

 A potential effect of satellite divergence is that it can drive coevolutionary changes in satellite-binding proteins within the pure species [21, 54]. According to this model, the sets of satellites and their binding proteins will evolve independently from those of different species. A consequence of these independent evolutionary trajectories is that a diverged protein from one species may not properly bind a satellite variant of another species in the hybrid background. This loss-of-function effect may occur particularly in cases in which satellite-binding proteins from only one parental species are expressed in hybrids, such as proteins encoded by X-linked genes in hemizygous males or proteins that are maternally contributed in the egg cytoplasm. Similar effects might also be expected to result in cases where a protein from one species is expressed at low levels or not at all so that satellite DNA is insufficiently packaged. Such a case has not yet been demonstrated in hybrids, but is a formal possibility and might resemble chromatin defects caused by mutational loss of GAGA factor in *D. melanogaster* [52]. Alternatively, deleterious gain-of-function interactions may occur, such as if a satellite-binding protein from one species associates inappropriately either with a diverged or functionally unrelated satellite or with a chromatin-modifying enzyme of another species.

 Compelling evidence of a satellite DNA/protein incompatibility was revealed through studies of the Odysseus-site homeobox (OdsH) protein in Drosophila hybrids. Crosses between *D. simulans* males and *D. mauritiana* females produce F1 hybrid males that are sterile. Interspecies cloning strategies identified *D. mauritiana* OdsH (OdsHmau), located on the X chromosome of this species, as a causal locus [55]. Although its function is unknown, OdsH is homologous to Unc-4, a known transcription factor, and is expressed in the apical end of the testes where the mitotic divisions preceding meiosis occur [56, 57]. Transgenic analysis revealed functional divergence between OdsH orthologs and the satellite DNA sequences to which it binds in each of these species. When expressed transgenically in *D. simulans* cells, OdsHsim and OdsHmau associated with similar satellite DNA regions on the X and 4th chromosomes [58]. However, OdsHmau bound to many additional regions on the *D. simulans* Y chromosome [58]. The specific amino acid changes between OdsH orthologs that give rise to their different binding patterns are not known, although substantial sequence divergence was discovered in the OdsH DNA-binding homeodomain [55]. OdsHmau recognizes only a small region of satellite DNA on the *D. mauritiana* Y-chromosome, suggesting that the sequences to which it binds have undergone expansion across the *D. simulans* Y chromosome [58]. Thus, interspecies divergence of both OdsH and its associated satellite DNAs appears to underlie these different binding patterns between *D. simulans* and *D. mauritiana*.

 It is currently unclear if hybrid sterility in this case results directly from differential OdsH binding to Y chromatin or to malfunction of an additional role of OdsH in the male germ line. However, several observations support the former possibility. First, deletion of the OdsH gene in *D. melanogaster* has little or no measurable effects on male fertility, demonstrating that OdsH is not an essential gene [56]. Second, the *D. simulans* Y becomes abnormally de-condensed in the presence of OdsHmau [58]. This effect could prevent the other chromosomes from segregating properly in the divisions preceding meiosis, thus leading to improper formation of sperm.

 How might OdsHmau induce Y decondensation? One possibility is that this protein may bind satellites on the *D. simulans* Y that it normally binds on the *D mauritiana* Y, but expansion of these sequences in the former species may lead to a chromosomal overloading of OdsHmau. Alternatively, OdsHmau may associate with expanded sequences on the *D. simulans* Y that are distinct from those that it normally binds in *D. mauritiana*. In either case, high concentrations of OdsHmau may disrupt normal localization of other essential chromatin proteins. Identification of OdsH polymorphisms that cause differential DNA binding, and the specific satellite DNA sequences and other chromatin proteins that OdsH interacts with in each species, will be helpful in exploring these possibilities.

## 4. Alteration of Chromatin Structure II: Heterochromatin-Related Effects

Another potential effect of satellite divergence in hybrids is disruption of heterochromatin. This term describes the exceptionally dense form of chromatin that packages satellites and other highly repetitive sequences during interphase (for a full review, see [59]). Two primary molecular features that define heterochromatin and govern its compact nature are (i) specific posttranslational Histone modifications and (ii) a small set of associating non-Histone proteins. The basic unit of chromatin is the nucleosome, consisting of DNA wrapped around an octamer of the Histone proteins H2A, H2B, H3, and H4. In heterochromatin, the C-terminal “tail” of Histone H3 carries methyl groups on Lysine residues 9 and 27. Added by Histone Methyltransferases (HMTs), these methyl groups serve as binding sites for non-Histone proteins such as the heterochromatin protein 1 (HP1) and its protein family members [60, 61]. It is believed that the association of HP1 with nucleosomes leads to the compact nature of heterochromatin [62, 63]. In addition to binding methylated Histone H3, HP1 also binds SU(VAR)3-9, a HMT, thereby recruiting this enzyme to chromatin where it can insure methylation of Histone H3 [64, 65]. Thus, the interactions of these proteins with one another and with the nucleosomes constitute a self-regulatory system that maintains the heterochromatic state, which can be epigenetically transmitted through cell lineages.

**Figure 2:**
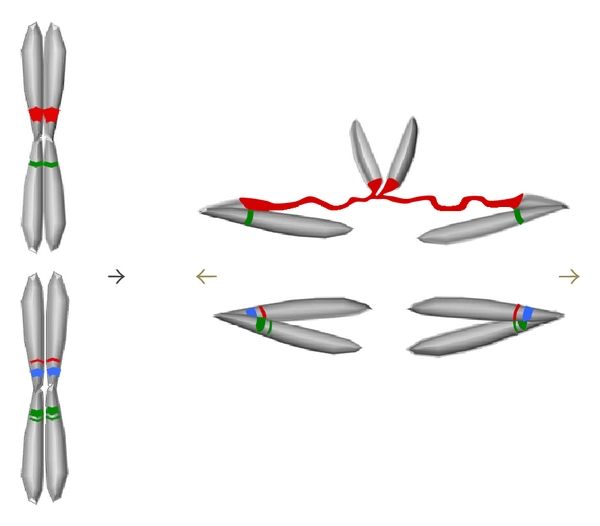
Disruption of mitotic chromosome segregation in hybrid embryos caused by satellite chromatin defects. Chromatid pairs line up at the metaphase plate for segregation at anaphase (left of arrow). The top chromatids fail to segregate due to defective chromatin structure of the red satellite block (right of arrow). This phenotype is analogous to that involving the 359-bp satellite block in *D. melanogaster*/*D. simulans* hybrid embryos [1] and results from an incompatibility between a *D. melanogaster*-specific satellite and a putative chromatin-related factor in the *D. simulans* egg cytoplasm.

Support for the idea that satellite DNA divergence can disrupt heterochromatin stems from studies of the *D. melanogaster Zygotic hybrid rescue* (*Zhr*) locus. Crosses between wild type *D. melanogaster* males and *D. simulans* females produce hybrid daughters that die during the cleavage divisions of early embryogenesis [66]. Previous genetic studies mapped a causal locus, *Zhr*, to a position near the centromere of the *D. melanogaster* X-chromosome [67]. Based on these and other genetic experiments [68, 69], it was proposed that *Zhr* consists of repetitive sequences in this region, a novel idea given that many of the known loci involved in reproductive isolation are protein-coding genes [55, 70–72]. More recent cytological analyses have supported this idea, demonstrating the presence of highly stretched region of 359-bp satellite DNA located on the *D. melanogaster* X during anaphase of mitosis in dying hybrid embryos [1]. This satellite region was found to prevent separation of the *D. melanogaster* sister X chromatids, inducing chromosome bridges and mitotic arrest (Figure 2).

 Two specific findings support the idea that these defects are due to improper heterochromatin formation. First, Topoisomerase 2 (Top2) was found to accumulate abnormally on the stretched 359-bp satellite block [1]. In addition to its enzymatic role in relieving supercoiled DNA, Top2 is a structural chromatin protein [73, 74]. In *D. melanogaster*, this protein is normally enriched on 359-bp satellite DNA at interphase and becomes evenly distributed across the chromosomes during mitosis [1]. In hybrids, however, Top2 remains abnormally localized to 359-bp satellite DNA throughout the cell cycle [1]. It is unlikely that *D. simulans* Top2, which is the only form present in the hybrid maternal cytoplasm, is the proximal cause, since this protein is highly conserved between *D. melanogaster* and *D. simulans* [1]. Moreover, hybrid females of the reciprocal cross are fully viable. Although only *D. melanogaster* Top2 is present in the egg cytoplasm of these individuals, *D. simulans* Top2 is expressed during later developmental stages while in the presence of the 359-bp satellite block, without deleterious effect.

 Second, the observed chromosomal defects occur at the developmental period when heterochromatin forms. In Drosophila, heterochromatin formation is marked by visible changes in chromatin density during early embryogenesis. The first 14 rounds of mitosis in this organism occur in a common cytoplasm derived from the egg before the nuclei individualize through the acquisition of their own plasma membranes [75]. These early divisions proceed under the control of factors present in the maternal cytoplasm until the beginning of zygotic gene expression, which occurs during mitotic divisions 12–14. Heterochromatin formation is marked by the appearance of dense regions of chromatin known as chromocenters during mitotic divisions 9-10 [76, 77]. It is precisely during these divisions when the first chromosome bridges appear in hybrid female embryos [1].

 Why might heterochromatin of the 359-bp satellite block fail to form in hybrids? One possibility is that some component(s) of the general heterochromatin machinery present in the *D. simulans* maternal cytoplasm are incapable of recognizing this *D. melanogaster*-specific satellite block. Although there is some precedence for this scenario in other systems [78], it is unlikely in this case for several reasons. First, the chromosome bridges in hybrid embryos appear during mitotic cycles 9-10, before HP1 and methylation of Histone H3 normally appear on the chromocenters [77]. Another general heterochromatin protein, SU(VAR)3-3, which is a homolog of the yeast demethylase LSD1, was recently shown to form foci in interphase nuclei as early as mitotic cycle 8, before bridge formation [79]. To our knowledge, however, this protein has not yet been examined for involvement in hybrid lethality. Second, the known protein components and posttranslational modifications to Histone H3 in heterochromatin, with few exceptions, are highly conserved from yeast to vertebrates [80]. This pattern stands in sharp contrast to the wide range of different satellite DNA sequences that exists within the genomes of most individual eukaryotic species, in all of which the heterochromatin machinery must properly package the entire sets of these sequences. It is, therefore, unlikely that the 359-bp satellite block poses challenges to the general heterochromatin machinery encoded by *D. simulans*.

 An alternative explanation may involve small, noncoding RNAs. Studies in *S. pombe* demonstrated that small RNAs derived from centric and pericentric repeats and the proteins that produce these small RNAs are essential for normal heterochromatin structure and centromere function [81]. It was proposed that these small RNAs facilitate heterochromatin formation and maintenance by recruiting the heterochromatin machinery to their complementary sequences for proper packaging. Experimental evidence for this model has since been documented in a number of additional organisms including *Arabidopsis thaliana* and *D. melanogaster* [82–85]. Small RNAs derived from the 359-bp satellite have been detected in the maternal cytoplasm of young *D. melanogaster* embryos [84, 85]. It was proposed that these small RNAs facilitate heterochromatin formation of the 359-bp satellite block in *D. melanogaster* [1, 82–84]. Moreover, the lack of the 359-bp small RNAs in the *D. simulans*-derived maternal cytoplasm of lethal hybrids may lead to mispackaging of this satellite block [1, 86]. One appeal of this model is that it takes into account the specificity of the observed defects, which appear confined to the 359-bp satellite block; all other sequences in hybrids appear normally packaged [1]. The fact that only this satellite block exhibits packaging defects in hybrids may be due to its large size, comprising nearly one half of the pericentric region on the *D. melanogaster* X. Other satellite DNAs unique either to *D. melanogaster* or *D. simulans* may incur problems in heterochromatin packaging but they may not be present in enough copies to alter chromosome segregation.

 Finally, the effects of 359-bp satellite DNA in hybrids may be tied to heterochromatin through parental imprinting. Best studied in mammalian eukaryotes, imprinting is a phenomenon that results in differential expression of certain genes when inherited from either the mother or father. In Drosophila, parental imprinting does not affect protein-coding genes, but instead involves the heterochromatic regions of the X- and Y-chromosomes (reviewed in detail in [87]). Imprinting effects in flies include differential levels of silencing of visible genetic markers that are located near these particular regions of heterochromatin. For example, the *scute* gene, located near the pericentric heterochromatin of the inverted X chromosome, *In *(1) *sc* ^8^, is expressed at lower levels when paternally inherited compared to transmission from the mother [88, 89]. Similar parental effects of reporter genes located within Y heterochromatin have also been observed [90, 91]. The nature of heterochromatic imprinting is not understood but may involve sex-specific differences in H3K9 methylation of heterochromatin that are established during gamete formation and/or early development [87].

 It is possible that the imprint of specific heterochromatic regions like the 359-bp satellite block may not be properly “interpreted” by the *D. simulans* maternal cytoplasm, resulting in the observed heterochromatin defects of this satellite in hybrids. One possible scenario is that the *D. simulans* cytoplasm fails to recognize *D. melanogaster*-specific Histone methylation or another unknown epigenetic mark on this satellite, which might be needed for proper heterochromatin packaging. Currently the Histone methylation state of the 359-bp heterochromatin has not been studied in hybrid embryos. However, a prediction based on the above hypothesis is that transmission of the 359-bp satellite block through the *D. simulans* maternal cytoplasm would result in suppression of packaging defects. Consistent with this prediction is the fact that hybrid females of the reciprocal cross, between *D. melanogaster* females and *D. simulans* males, are completely viable. In this case, the 359-bp satellite block should be imprinted maternally through the *D. melanogaster* egg cytoplasm. However, it is important to point out that the viability of reciprocal female hybrids is also consistent with mechanisms involving diverged satellite-binding proteins or repeat-derived small RNAs outlined above.

## 5. Release of Meiotic and Postmeiotic Drive Systems

**Figure 3:**
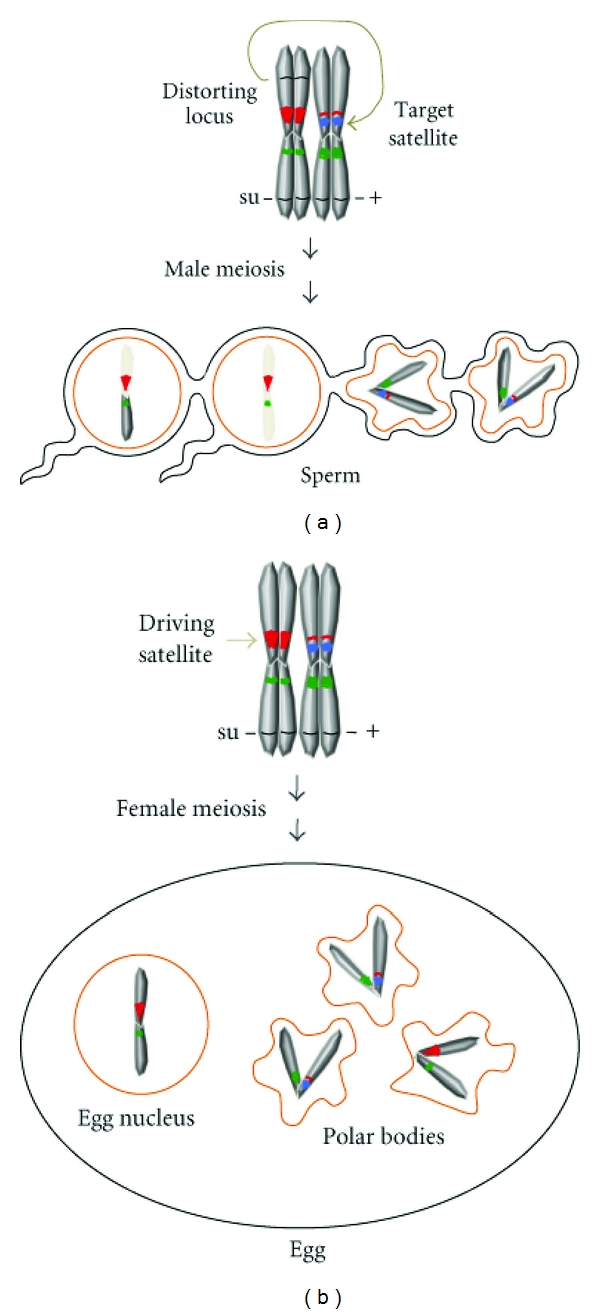
Segregation distortion in hybrid animals. (a) Postmeiotic release of segregation distortion in hybrid males. A recessive suppressor of distortion (su) in one species becomes inactive in the heterozygous hybrid. This allows the distorting locus to target a satellite block on the chromosomes of the other species (top). This effect results in spermatid bundles (bottom) in which spermatids inheriting the targeted chromosome fail to individualize. The spermatids carrying the chromosome with the distorting locus develop normally. (b) Release of meiotic drive in hybrid females. A recessive suppressor becomes heterozygous in the hybrid female. This enables a chromosome from one species, which carries a “selfish” satellite, to outcompete the homologous chromosome from the other species. As a result, the egg nucleus will carry a chromosome with the selfish satellite, and chromosomes lacking these satellites will end up in the unused polar bodies.

Under normal circumstances, homologous chromosomes are segregated equally into gametes. However, some loci are capable of altering chromosome segregation during or after meiosis in order to selfishly transmit themselves at unusually high frequencies. In these cases, satellite variants can be either the targets of drive or the driving elements themselves (Figure 3).

 One well-known example of postmeiotic drive involving satellites is the Segregation Distorter (SD) system in *D. melanogaster*. The selfish component of SD is a duplicated gene on chromosome 2 encoding a truncated RanGAP protein [92]. In males that are heterozygous for this mutant allele, *Sd*, and the wild type allele, *Sd* ^+^, the entire half of the spermatids containing the *Sd* ^+^ allele exhibit chromosome condensation defects and they fail to mature. Thus, only chromosomes carrying the selfish *Sd* allele are transmitted. *Sd* does not target the *Sd* ^+^ allele itself, but instead, a closely linked satellite block consisting of a 240-bp monomer known as Responder (*Rsp*). *Rsp* satellite blocks consisting of ~200 to 3,000 or more monomers (termed Responder-sensitive or *Rsp* ^S^) are targeted, whereas smaller blocks (Responder-insensitive or *Rsp* ^I^) are unaffected [25]. This effect favors *Sd* since it is linked to *Rsp* ^I^ blocks, whereas *Sd* ^+^ is often linked to *Rsp* ^S^ blocks. It is currently not known how *Sd* targets *Rsp* ^S^ satellite blocks at the molecular level, but may involve mislocalization of *Sd*-encoded RanGAP that leads to chromosome decondensation through a number of possible mechanisms [86, 93, 94].

 Distorting loci like *Sd* may eventually harm individuals and populations, such as when distorters are closely linked to deleterious alleles, or if distortion involves the sex chromosomes, thus affecting the sex ratio balance in populations, respectively. As a counter, unlinked suppressors of distortion may evolve. Suppressors are effective until mating occurs with individuals that do not carry them, in which case suppression is lost and the driving phenotype is unleashed (Figure 3(a)). In agreement with this idea, several different masked distortion systems have been identified through both interstrain and interspecies Drosophila crosses [94, 95]. In these cases, the targets of distortion are not known, but may involve species-specific satellites since defects in spermatogenesis are highly similar to those present in *Sd* distortion [94].

 Distorting loci can also be the satellites of centromeres or their adjacent regions. One process in which these sequences are thought to be particularly prone to non-Mendelian segregation is female meiosis. This is due primarily to the fact that meiosis in females is asymmetric; four meiotic products are produced but only one becomes the egg's hereditary material, while the other three products form polar bodies and are eliminated. It has been proposed that certain centromeric satellite variants can take advantage of this asymmetry by outcompeting other sequences for extraordinarily high rates of transmission into the egg's nuclear material (Figure 3(b)) [96–98].

 Non-Mendelian segregation of certain alleles during female meiosis has been detected genetically in a number of organisms [99–102]. However, the most direct evidence for meiotic drive of repetitive elements stems from one study in Mimulus (monkeyflower) species hybrids. Crosses between *Mimulus guttatus* and *M. nasutus* resulted in release of a suppressed meiotic driver locus on the *M. guttatus* chromosome 2 that approaches transmission of 100% [103]. Genetic and cytological mapping revealed that the driving element is located in or immediately adjacent to the centromere, consistent with the possibility that the element is a satellite [102]. Interestingly, this driving allele is associated with a fitness cost in hybrid males. In the pure species, such deleterious effects may prevent selfish elements from reaching fixation before driving suppressors can evolve. Future molecular and cytological studies in this system will help to test existing models that predict how meiotic drive might occur at the molecular and cellular levels [98, 104].

## 6. Satellite Divergence and the Dobzhansky-Bateson-Muller Model of Hybrid Incompatibility

Early work by Dobzhanksy, Bateson, and Muller provided the foundation for a genetic model that explains the evolution of hybrid sterility and lethality [105]. The simplest form of this model involves a pair of loci, each of which has diverged functionally between sibling species. The products of these loci malfunction when expressed together in hybrids, leading to developmental defects that cause sterility or lethality. Such interspecies molecular interactions that reduce hybrid fitness are referred to as hybrid incompatibilities (HIs). Over the past decade, a number of HI loci have been identified. Some of these loci encode proteins [106]. It was proposed that HI loci encoding transcription factors cause large-scale misregulation of gene expression in *D. simulans*/*D. melanogaster* hybrids [70], although this was later shown to not be the case [107]. Other models implicate deleterious interactions between proteins encoded by HI loci [108]. In general, much remains to be uncovered mechanistically regarding the majority of HI cases that involve protein-coding genes.

 A number of studies discussed here have documented the negative effects of satellite divergence on chromosome behavior in hybrids. The results from these studies have demonstrated that satellites, like protein-coding genes, can operate as HI loci. The biology of satellites is complex, with a diverse array of associated factors including general and specific heterochromatin proteins, small RNAs, and epigenetically modified histones that are often developmentally regulated. This complexity offers researchers new ways to envision how HI might occur in hybrids and new HI candidates to test.

 At the core of the evolution of such HI cases may be a scenario in which rapidly evolving satellite sequences force their packaging or associating proteins to evolve equally rapidly in order to preserve chromosome function in the pure species. However, proteins—or perhaps other factors—adapted to satellites from one species may interact inappropriately with diverged satellites from another species in hybrids, thus causing HI. The complex nature of satellite heterochromatin is consistent with previous speculation that most HI interactions may be more complex than the two-locus model [109]. Reciprocally, however, the existence of satellite HI loci may also offer more simplified views of HI, such as an HI locus pair consisting of satellite DNA in one species and the absence of complementary small RNAs in the other species. Indeed, satellite DNA may even be regarded as a special type of HI locus because it can direct its own packaging by generating small RNAs, thus operating as both the cause and suppressor of HI [86].

 Given the functional involvement of satellites in chromosome dynamics and their evolutionarily labile nature, it is no surprise that these sequences make up a common type of reproductive isolating locus. Further exploration will, no doubt, be challenging due to difficulties in manipulating satellite sequences and the epigenetic states of heterochromatin, but they will progressively reveal a more detailed picture of how these hybrid incompatibilities occur at the molecular level.
